# Antigen-Binding Properties of Monoclonal Antibodies Reactive with EBNA1 and Use in Immunoaffinity Chromatography

**DOI:** 10.1371/journal.pone.0004614

**Published:** 2009-02-26

**Authors:** Sarah J. Duellman, Richard R. Burgess

**Affiliations:** McArdle Laboratory for Cancer Research, University of Wisconsin-Madison, Madison, Wisconsin, United States of America; Institut Pasteur Korea, Republic of Korea

## Abstract

Epstein-Barr virus (EBV) nuclear antigen 1 (EBNA1) was overexpressed and purified from *Escherichia coli*. Mouse monoclonal antibodies (mAbs) were prepared that react with EBNA1. Eleven high affinity mAbs were recovered. Nine mAbs are isotype IgG (all subisotype IgG_1_) and two mAbs are isotype IgM. All mAbs react strongly with EBNA1 in an ELISA assay while only one mAb (designated 1EB6) fails to react in a Western blot assay. The epitopes for these mAbs were mapped to seven different regions, providing good coverage of the entire EBNA1 protein. The mAbs had differing affinity for an EBNA1/DNA complex with four mAbs able to supershift the complex completely. All mAbs can immunoprecipitate EBNA1 from *E. coli* overexpressing EBNA1. A modified ELISA assay, termed ELISA-elution assay, was used to screen for mAbs that release EBNA1 in the presence of a low molecular weight polyhydroxylated compound (polyol) and a nonchaotropic salt. MAbs with this property, termed polyol-responsive (PR)-mAbs, allow gentle elution of labile proteins and protein complexes. Four mAbs are polyol-responsive with two showing usefulness in gentle immunoaffinity chromatography. Purification with these PR-mAbs may be useful in purifying EBNA1 complexes and elucidating EBNA1-associated proteins. This panel of anti-EBNA1 mAbs will advance the study of EBV by providing new tools to detect and purify EBNA1.

## Introduction

Epstein-Barr virus (EBV) is a gamma-herpesvirus that is the causative agent of infectious mononucleosis and a risk factor in a variety of malignancies, including Burkitt's lymphoma and nasopharyngeal carcinoma [1], [2]. EBV nuclear antigen 1 (EBNA1) is the only viral protein found in all EBV-related malignancies [3]. EBNA1, a 641-amino acid protein, plays an important role in maintaining EBV in the host cell and has been implicated in host cell immortalization. It is the only viral factor required in *trans* for efficient replication of the EBV genome during latency [3] and regulates transcription at multiple viral promoters [4]–[6].

The N-terminus of EBNA1 consists primarily of a 239-amino acid domain comprised of a Glycine-Glycine-Alanine (GGA) repeat region. An EBNA1 derivative (referred to as 1553), encoding only fifteen residues from the GGA repeat region, maintains the ability to support replication and transcription in cell culture [7] and the ability to immortalize B cells [8]. Amino acids 64–89 comprise a transcriptional activation domain [9]. The C-terminus of EBNA1 contains a dimerization domain and a DNA binding domain. EBNA1 also contains two linking regions (LR1 and LR2), which allow EBNA1 dimers bound to DNA to associate with other bound EBNA1 dimers and thereby loop intervening DNA sequences *in cis* or link two DNA molecules *in trans* [10], [11].

Though the protein domain structure is well described, many fundamental aspects of EBNA1 biology remain poorly understood. We have recently developed vectors and procedures for readily expressing and purifying various EBNA1 constructs in *E. coli*, described in [12], allowing us to generate a diverse library of EBNA1-specific murine monoclonal antibodies (mAbs). Mice were injected with increasing amounts of EBNA1 protein, and the spleen cells were fused to myeloma cells to generate hybridoma cell lines. Four fusions have been completed, resulting in eleven high affinity mAbs. Most mAbs are subisotype IgG_1_ molecules and react with epitopes located throughout the EBNA1 protein. We screened the mAbs for specific reactivity to EBNA1 by enzyme-linked immunosorbent assay (ELISA) and Western blot analysis, and analyzed their ability to supershift an EBNA1/DNA complex.

We tested each mAb for its ability to immunoprecipitate (IP) EBNA1 from whole cell extract. Although immunoaffinity chromatography (IAC) is a powerful purification technique, it usually requires a high concentration of denaturing reagents and/or extreme pH to dissociate the antigen-antibody interaction. As a result, inactive, denatured proteins are recovered and protein complexes are difficult to maintain. Our lab has pioneered work using polyol-responsive mAbs (PR-mAbs) in IAC. PR-mAbs allow gentle elution through the use of a low molecular weight polyhydroxylated compound (polyol), like propylene glycol (PG) or ethylene glycol, and a nonchaotropic salt, like NaCl or ammonium sulfate (AS). Typically, only approximately 10–20% of all mAbs tested have this property [13].

PR-mAbs are especially useful in purifying proteins that retain biological activity and structural integrity. PR-mAbs allow purification in sufficient yield, purity, and homogeneity to allow crystallization of protein [14]. If the protein of interest is involved in protein complexes, PR-mAbs provide a method of gently eluting the protein resulting in recovery of functional proteins and multi-subunit complexes that are structurally intact and biologically active (for reviews see [15], [16]). Many groups have been able to isolate protein complexes using PR-mAbs and determine the interacting partners of their protein of interest [13], [14], [17]–[19]. Four anti-EBNA1 mAbs showed polyol-responsiveness in our ELISA-elution assay with two shown to be useful in purifying EBNA1 in one chromatographic step using PR-mAb IAC technology.

## Materials and Methods

### Reagents and buffers

All reagents were obtained from Sigma Chemical (St. Louis, MO) unless otherwise noted. TE buffer contains 50 mM Tris-HCl and 0.1 mM EDTA, pH 7.9. BLOTTO contains 1% nonfat dried milk in phosphate-buffered saline (PBS).

### Plasmids

**Figure 1:**
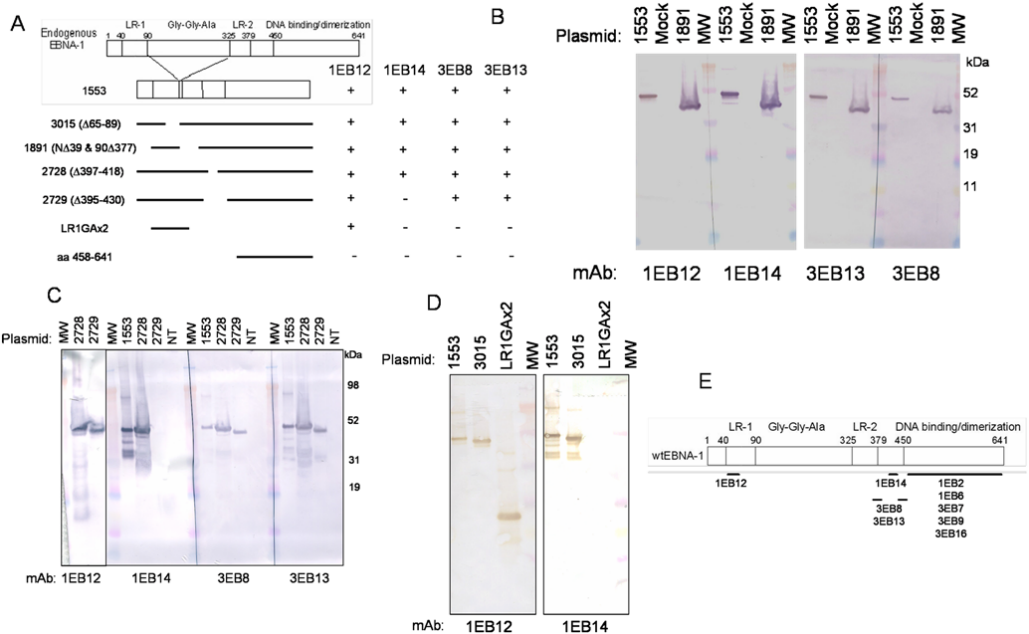
Epitope mapping of the N-terminal anti-EBNA1 mAbs. A) The constructs used to analyze mAb reactivity. Full length EBNA1 is shown at the top, numbered according to the B95-8 EBV strain. LR-1 and LR-2 (linking regions 1 & 2) are designated, as well as the GGA repeat region between amino acids 90 and 325. The C-terminus is largely composed of the DNA-binding and dimerization domain. 1553 encodes functionally wildtype EBNA1, but lacks the majority of the GGA stretch. The remaining EBNA1 derivatives are derived from the 1553 construct and thus carry the GGA deletion. B) 1891 was transfected into 293 cells and whole cell extract was analyzed by Western blot. The first lane in each series contains purified 1553, the second lane is untransfected 293 whole cell extract (mock), and the third lane is whole cell extract from cells transfected with the 1891 plasmid. Equal concentrations of purified mAbs were used to probed the Western blots. C) Plasmids 2728 and 2729 were transfected into 293 cells and whole cell extract was analyzed by Western blot. NT, not transfected. Equal concentrations of purified mAbs were used to probe the Western blots. D) Purified EBNA1 derivatives were analyzed by Western blot for mAb reactivity. Equal concentrations of mAb 1EB12 or mAb 1EB14 were used to probe the Western blots. E) Summary of the anti-EBNA1 mAb epitopes. The schematic shows endogenous EBNA1 protein, numbered according to the B95-8 strain of EBV, and the domain structure. The lines represent the various epitope regions and the mAbs that interact in that area are written below. MW is molecular weight.

1553 encodes a fully functional EBNA1 variant that contains 15 residues of the GGA repeat present between amino acids 90 and 325 of EBNA1 encoded by the B95-8 strain of EBV [20]. All other derivatives used in this study also contain this deletion. 1553 will be referred to as EBNA1. See schematic in Fig. 1A. 3015 additionally lacks amino acids 64–89. Both plasmids were ligated into the pET22b expression vector (Novagen, Madison, WI) as described in [12]. 1891 contains two deletions: 1) N-terminus to amino acid 39 and 2) amino acid 90 to 377. This derivative retains LR1, nuclear localization sequence (NLS), and the DNA-binding and dimerization domains. 2728 and 2729 lack amino acids 397–418 and 395–430, respectively. All of the plasmids listed above were gifts from Dr. Bill Sugden. LR1GAx2 is a construct that contains two copies of the LR1 domain with 18 amino acids from the GGA repeat region in the center. This construct is cloned into the pET28b expression vector (Novagen). 1553, 3015, and LR1GAx2 were all expressed in *E. coli* as described in [12].

### Cells

293 cells are derived from human embryonic kidney cells [21] and were grown in Dulbecco's modified Eagle's medium (Invitrogen) supplemented with 10% fetal bovine serum, 200 U/ml penicillin, and 200 µg/ml streptomycin. All cells were grown at 37°C in a humidified 5% CO_2_ atmosphere.

### Transfections

Plasmids 1891, 2728, and 2729 were transiently transfected into 293 cells. 5 µg of DNA and 5 µg of empty vector DNA were combined in 500 µl Opti-Mem (Invitrogen) and mixed with 40 µg polyethyleneimine (PEI) in 500 µl Opti-Mem. The solution was added to 10 ml of cells, and incubated at 23°C for 20 min. Cells were allowed to grow 48 h at 37°C and harvested.

### Hybridomas and mAbs

For the isolation of hybridomas that produce EBNA1-specific mAbs, Ni-NTA-purified recombinant EBNA1 was prepared as described in [12] and injected into Balb/c ByJ mice (Jackson Laboratory, Bar Harbor, ME) according to the following schedule: four female mice were each injected on day 1 with 5 µg, on day 14 with 10 µg, and on day 28 with 20 µg. The first injection was contained in Freund's complete adjuvant, and subsequent injections were contained in Freund's incomplete adjuvant. Each injection (100 µl) was administered subcutaneously (SQ) and intraperitoneally (IP). Animals were bled on day 1 to obtain the pre-immune sera for a negative control and day 42 to test for reactivity with EBNA1 antigen in an enzyme-linked immunosorbent assay (ELISA). All sera showed a titer of greater than 1∶6400 when assayed by ELISA. One mouse was injected 90 days after the third injection with 60 µg EBNA1 contained in PBS, administered IP. Three days later, the animal was sacrificed, and the spleen cells were fused with NS-1 and SP2/0 myeloma cells using standard hybridoma techniques [22]. Fusions were screened by ELISA and Western blot assay. Hybridomas were cloned twice by limiting dilution. Isotyping was performed using an ELISA-based kit (HyClone, Logan, UT). All animal protocols were approved by the University of Wisconsin-Madison School of Medicine and Public Health Animal Use and Care Committee.

### Purification of mAbs

IgG_1_ mAbs were grown in Celline flasks (IBS Integra Biosciences, Chur, Switzerland) according to manufacturer instructions and purified as follows. To remove albumin, mAb sample was brought to 45% saturated ammonium sulfate, mixed on ice for 20 min, and incubated at 4°C for 18 h. The sample was collected by centrifugation (12,000 g for 20 min at 4°C). The pellet was resuspended in antibody buffer (50 mM Tris-HCl, 25 mM NaCl, pH 6.9) and dialyzed into antibody buffer at 4°C for 18 h. Any precipitate was removed and the supernatant was applied to a DEAE cellulose (Whatman, Maidstone, England) column equilibrated in antibody buffer at 23°C. The flowthrough contains purified mAb. MAb 1EB14 did not flow through the DEAE column, but eluted from the column with 500 mM NaCl. MAb 1EB14 was additionally purified using a 15-ml PorosHQ50 anion exchange chromatography column. The sample was diluted to 25 mM NaCl, loaded, washed with 25 mM NaCl, and eluted over a 25 mM–1 M NaCl salt gradient in TE buffer.

### Preparation of mAb-Sepharose

mAb-Sepharose was prepared as described previously [13]. MAb was dialyzed into coupling buffer (0.1 M NaHCO_3_, 0.5 M NaCl, pH 8.3) overnight at 4°C. Purified mAb (2.5 mg/ml swollen Sepharose) was conjugated to cyanogen bromide (CNBr)-activated Sepharose 4B. CNBr-activated Sepharose was re-swollen in 1 mM HCl and rinsed with coupling buffer. Sepharose and mAb were mixed end-over-end at 23°C for 2 h and collected on a sintered glass filter. MAb-Sepharose was mixed with blocking agent (6% ethanolamine in coupling buffer, pH 8.0) for 2 h at 23°C. Four sets of alternating washes of coupling buffer and acetate buffer (0.1 M sodium acetate, 0.5 M NaCl, pH 4.0) were used to remove non-covalently bound protein from the resin. MAb-Sepharose was stored in TE+0.02% NaN_3_ at 4°C. This resin can be used multiple times if treated and stored as described [15], [16].

### Preparation of mAb-rProteinA beads

MAbs were dialyzed into crosslinking buffer (50 mM sodium borate, 3 M NaCl, pH 9.0) and added to rProteinA-agarose beads (Repligen, Waltham, MA). The reaction proceeded with gentle shaking for 1 h at 23°C. The beads were washed with crosslinking buffer. 20 mM dimethyl pimelimidate-2HCl (DMP) crosslinker (Pierce, Rockford, IL) was added and incubated with the beads for 30 min at 23°C. 50 mM Tris-HCl, pH 7.9 was used to wash the beads and quench the reaction over a 2 h incubation at 23°C. The resin was stored in PBS+0.02% NaN_3_ at 4°C.

### ELISA-elution assay

ELISA-elution assays were performed as described [13], [15], [16], [19]. Microtiter plates were coated with 50 ng of EBNA1 in a 50 µl volume of PBS at 23°C with shaking for 2 h. The plate was blocked with 200 µl per well of BLOTTO at 4°C overnight. The plate was washed 3 times with PBST (PBS+0.1% Tween 20). Purified antibody diluted 1∶1000 in BLOTTO or undiluted cell supernatant from hybridoma cell culture was added to the wells and incubated for 1 h at 23°C. The plate was washed three times with PBST. Polyol elution buffer (TE buffer plus various combinations of propylene glycol and ammonium sulfate) was added to each well and allowed to incubate for 20 min. The plate was washed three times with PBST. Remaining primary antibody was detected using an enzyme-conjugated secondary antibody and the appropriate substrate.

### Electrophoresis and Western blotting

Proteins were separated by electrophoresis using 4–12% Bis/Tris NuPAGE polyacrylamide gels (Invitrogen). Western blots to detect EBNA1 expressed in *E. coli* were prepared as described previously [12], with a secondary antibody (goat anti-mouse IgG) conjugated to alkaline phosphatase and the 5-bromo-4-chloro-3-indolyl phosphate and nitroblue tetrazolium (BCIP/NBT) reagent as substrate. Western blots to detect EBNA1 from lymphoma cell lines used the secondary antibody goat anti-mouse IgG, human serum adsorbed, conjugated to horseradish peroxidase (KPL, Gaithersburg, MD) and enhanced chemiluminescent (ECL) substrate (Pierce) for detection. Prestained molecular weight markers (Multimark, Invitrogen) were included on all gels. MagicMark molecular weight markers (Invitrogen) were included on the gels for ECL detection.

### Immunoaffinity purification of EBNA1

To test the applicability of these mAbs to immunoprecipitate (IP) EBNA1 from *E. coli* whole cell extract, a frozen cell pellet (7.5 mg wet weight) from 5 mls of induced culture was resuspended in 100 µl TE+0.1 M NaCl. Cells were lysed by sonication (4 rounds of 30 s bursts on ice). Cell debris and inclusion bodies were removed by centrifugation (27,000 g for 20 min at 4°C). The soluble fraction was added to 40 µl of 50% (w/v) mAb-Sepharose slurry, previously equilibrated two times in 5 column volumes (CV) of TE+0.1 M NaCl. Cell extract and resin were mixed at 23°C for 1 h. The resin was washed twice with 5 CV TE+0.1 M NaCl and twice with 5 CV of TE+0.5 M NaCl. 1 CV of 2× sodium dodecyl sulfate (SDS) sample buffer plus heating at 95°C for 4 min was used to elute the bound proteins. Results were analyzed by Western blot using 2B4 rat anti-EBNA1 mAb [23] for the *E. coli*-expressed samples.

### Immunoaffinity purification of EBNA1 using polyol-responsive mAbs

Frozen cell pellet (1.5 g wet weight) from 1 L of induced *E. coli* culture was resuspended in 20 ml TE+0.1 M ammonium sulfate (AS). Cells were lysed by sonication (4 rounds of 30 s bursts on ice). Cell debris and inclusion bodies were removed by centrifugation (27,000 g for 20 min at 4°C). The soluble fraction was applied to a 4-ml column of either 1EB2- or 3EB7- Sepharose. The columns were washed with 2.5 CV of TE+0.1 M AS and either 2.5 CV of TE+40% propylene glycol (PG) for 1EB2 or 2.5 CV of TE+0.5 M AS for 3EB7. EBNA1 was eluted with TE+0.75 M AS+40% PG from both columns.

### Electrophoretic mobility shift assay

Plasmid 3304 was used to generate a 176-bp oligonucleotide encoding a 16-bp palindromic EBNA1 binding site of sequence GGTAGCATATGCTACC [24]. Five hundred femtomoles of the double-stranded DNA probe was end-labeled by T4 polynucleotide kinase (New England Biolabs, Ipswich, MA) with 2 pmol [γ-^32^P]ATP in a volume of 20 µl. Ten femtomoles of end-labeled probe was incubated with 40 ng EBNA1 in a total volume of 15 µl. The reaction was carried out in 1× EMSA buffer (20 mM Hepes, pH 7.6, 40 mM KCl, 1 mM MgCl_2_, 1 mM EDTA, and 10% glycerol) with 20 µg/ml poly(dI-dC) (GE Healthcare) for 15 min at 23°C. For the mAb supershift assays, 50 ng mAb was added, and the reactions were allowed to incubate at 23°C for an additional 15 min. The reactions were subjected to electrophoresis using a 3–8% Tris/acetate NuPAGE gel (Invitrogen) at 125 V at 4°C for 3.5 h. Gels were transferred to Whatman paper, dried at 80°C for 1 h, and analyzed by PhosphoImager Screen analysis using a Storm Imager (Molecular Dynamics, Sunnyvale, CA).

## Results and Discussion

### EBNA1 mAb generation

The *E. coli*-expressed EBNA1 protein was used to immunize mice to generate EBNA1-specific mAbs. Mice were injected with increasing amounts of EBNA1 protein, sacrificed, and the spleen cells were fused to myeloma cells to produce hybridoma cell lines. Four fusions were completed isolating 11 high affinity mAbs, two are isotype IgM and the remaining 9 are subisotype IgG_1_. Table 1 summarizes the characteristics of these EBNA1-reactive mAbs.

**Table 1:** Summary of anti-EBNA1 murine mAbs.

mAb	Isotype	Epitope	WB	WB'	ELISA	PR-mAb	IP	Supershift
1EB2	IgG1	451–641	Y	Y	1.1±0.04	Y^a^	++	−
1EB3	IgM	1–458	Y	Y	1.1±0.07	N	NYT	NYT
1EB6	IgG1	458–641	N	N	2.1±0.05	N	++	++
1EB12	IgG1	40–65	Y	Y*	1.4±0.04	N	++	−
1EB14	IgG1	418–430	Y	Y	1.4±0.13	N	++	++
2EB3	IgM	458–641	N	N	1.4±0.04	Y	NYT	NYT
3EB7	IgG1	458–641	Y	Y	1.9±0.08	Y	++	+
3EB8	IgG1	377–395 or 430–458	Y	Y	1.4±0.01	N	++	−
3EB9	IgG1	458–641	Y	Y	1.7±0.06	N	++	++
3EB13	IgG1	377–395 or 430–458	Y	Y	1.6±0.01	Y	+	+/−
3EB16	IgG1	458–641	Y	Y	1.9±0.13	N	++	++

WB: Western blot, WB': Western blot signal with endogenous EBNA1, ELISA: the average ELISA signal when adding equal concentrations of purified mAb to an EBNA1-coated well, PR-mAb: polyol-responsive mAb, IP: immunoprecipitation, Supershift: ability to bind to EBNA1/DNA complex and change its mobility during non-denaturing gel electrophoresis, NYT: not yet tested. Epitope was determined as in Fig. 1.

* particularly high affinity for endogenous EBNA1.

a responsive to salt alone.

The mAbs were screened for specific reactivity to EBNA1 by ELISA and Western blot analysis (Table 1). All mAbs reacted with full-length EBNA1 by Western blot, except mAb 1EB6 (data not shown). This mAb reacted strongly in the ELISA assay, but showed no affinity for EBNA1 on a Western blot. The structure of EBNA1 may be important to the binding of mAb 1EB6 since the ELISA assay retains more of the native structure than Western blot analysis. Also, the IgM mAb 2EB3 did not react specifically to EBNA1 on a Western blot, which is commonly seen with the IgM isotype.

MAbs were also tested for reactivity with endogenous EBNA1 from the 721 lymphoblastoid cell line (LCL) (data not shown). This EBNA1 protein carries the full GGA stretch and, presumably, post-translational modifications that may influence the binding of mAbs to the protein. Most mAbs did not have high enough affinity to detect the small amount of EBNA1 present in this sample. MAb 1EB12 had an especially strong interaction with endogenous EBNA1, followed by mAb 1EB14.

### Epitope mapping

Anti-EBNA1 mAbs were first tested for their ability to bind a C-terminal derivative of EBNA1, containing amino acids 458–641. MAbs 1EB6, 3EB7, 3EB9, and 3EB16 all bound this derivative by ELISA and/or Western blot analysis. MAb 1EB2 had an intermediate binding affinity for this derivative when compared to full-length EBNA1, as seen in ELISA (data not shown). However, mAb 1EB2 bound as strongly to a derivative encoding amino acids 451–641 as it did to full-length EBNA1. These data indicate that only a portion of the epitope for mAb 1EB2 is contained within amino acids 458–641 or additional N-terminal amino acids are necessary to stabilize the epitope. C-terminal mAb 1EB6 also maps to residues 458–641 by ELISA, but does not react in a Western blot suggesting it interacts with a different region within this peptide. It is likely that there are several different epitopes contained within this group; more testing of various C-terminal EBNA1 derivatives would be required to map these epitopes more precisely.

To narrow down the epitopes of the mAbs that bind in the N-terminal half of EBNA1 (1EB12, 1EB14, 3EB8 and 3EB13), Western blot analysis was performed on a variety of EBNA1 derivatives. These constructs are shown in Fig. 1A. All mAbs reacted with 3015, which carries a deletion of unique region 1 (UR1), amino acids 65–89. All mAbs also reacted with 1891 which carries two deletions: 1) N-terminus to amino acid 39 and 2) amino acid 90 to 377 (Fig. 1B). MAbs 1EB12, 3EB8, and 3EB13 reacted with both 2728, which carries a deletion of amino acids 397–418, and 2729, carrying a deletion of amino acids 395–430 (Fig. 1C). MAb 1EB14, however, only reacted with 2728, not 2729. The epitope for mAb 1EB14 is most likely contained within amino acids 418–430, however, the deletion of amino acids 395 and 396 may remove an essential residue of the mAb 1EB14 epitope. MAbs 3EB8 and 3EB13 react either with amino acids 377–395 or 430–458.

mAb 1EB12 is unique in that it is the only mAb to bind LR1GAx2, a derivative that encodes two LR1 peptides (amino acids 40–90) bordering an 18-amino acid stretch of the GGA repeat (Fig. 1D). Since mAb 1EB12 interacts with 1891 (lacking any sequence from the GGA repeat) and 3015 (deleting half of LR1), the epitope must be located within amino acids 40–65. EBNA1 is methylated on the arginines contained within this stretch of amino acids [25]. Since mAb 1EB12 has a much stronger reactivity to endogenous EBNA1 compared to the rest of the mAbs while the reactivity to *E. coli*-expressed EBNA1 is similar to the rest of the mAbs, it is possible that the modification of these arginine residues increases the affinity of mAb 1EB12 for its epitope. MAb 1EB3 has not been tested extensively, but its epitope is contained within amino acids 1–458.

This panel of anti-EBNA1 mAbs provides good coverage of the entire EBNA1 protein. A summary of the epitopes for the anti-EBNA1 mAbs is shown in Fig. 1E and Table 1.

### Ability of mAbs to bind EBNA1/DNA complex

**Figure 2:**
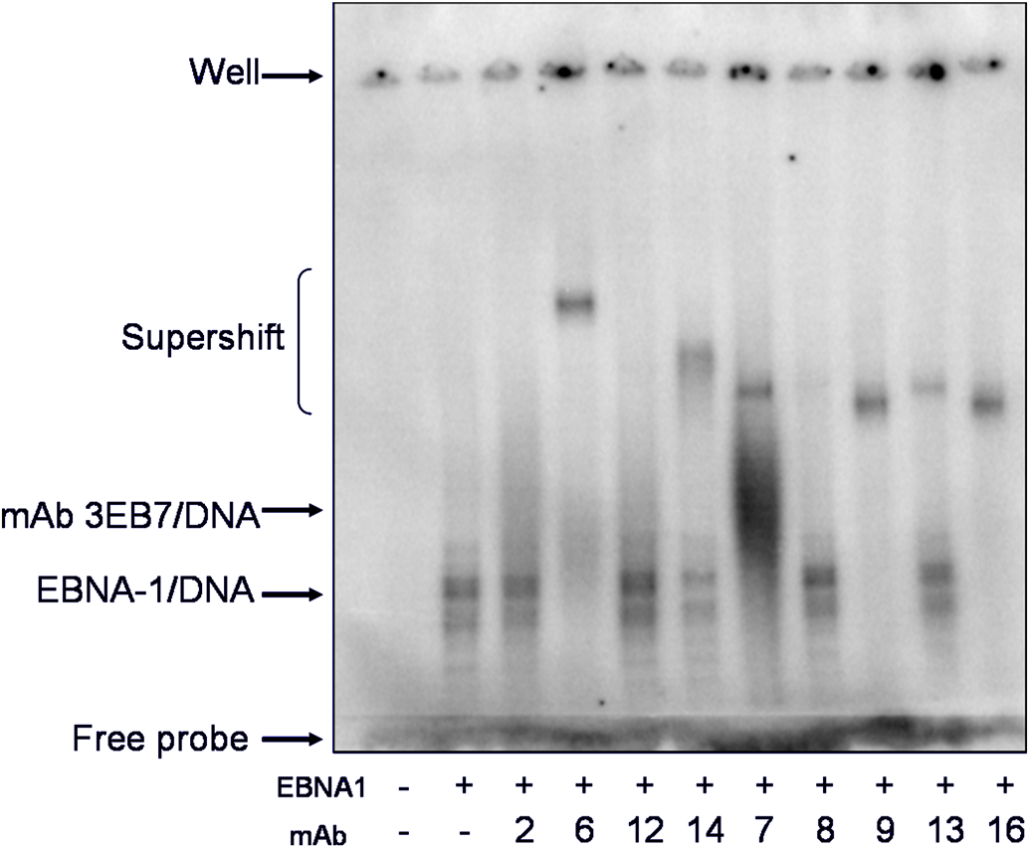
Ability of mAbs to bind EBNA1/DNA complex. MAbs were tested by EMSA to determine binding efficiency to a pre-formed EBNA1/DNA complex. Purified EBNA1 protein was bound to a ^32^P-labelled oligonucleotide encoding one palindromic EBNA1 binding site. Equal concentrations of purified mAb were added to each reaction and allowed to incubate. The ability of the mAbs to shift the EBNA1/DNA complex was analyzed by gel electrophoresis. The first lane does not include any protein. The second lane contains only DNA and EBNA1. MAbs are listed by the last number in their name.

To test the ability of these mAbs to bind to an EBNA1/DNA complex, an electrophoretic mobility shift assay (EMSA) was utilized (Fig. 2). The DNA probe contained one high affinity binding site for EBNA1. An EBNA1/DNA complex was formed and then the mAbs were added. The same experiment was done first forming the EBNA1/mAb complex and then adding DNA, but the results were the same suggesting that either the half-life of these interactions is shorter than the incubation time allowed or the formation of these complexes does not interfere with each other such that everything can bind simultaneously.

The EBNA1/DNA complex alone migrated and formed a doublet that was consistently seen. The doublet may have been a result of DNA and/or EBNA1 sample heterogeneity. MAbs 1EB6, 1EB14, 3EB9, and 3EB16 were able to supershift the EBNA1/DNA complex very strongly, with mAbs 1EB6, 3EB9, and 3EB16 shifting all of the EBNA1/DNA complex. MAbs 3EB13 and 3EB7 were able to supershift the complex to an intermediate level. MAb 3EB7 had a nonspecific interaction with the DNA probe (data not shown) that resulted in the dark smear below the supershift band. MAbs 1EB2 and 1EB12 showed a smear above the EBNA1/DNA complex bands suggesting that the mAbs are able to bind the complex weakly, dissociating randomly throughout electrophoresis, therefore resulting in a shifted protein smear. Incubation with mAb 3EB8 resulted in a very light supershift band.

### Immunoprecipitation of EBNA1

**Figure 3:**
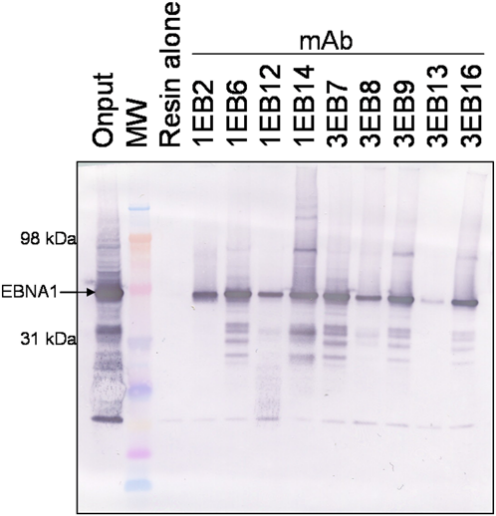
Immunoprecipitation of EBNA1. *E. coli*-expressed EBNA1 (∼50 kDa) was isolated from whole cell extract by mixing with equal quantities of mAb-rProteinA-agarose resin. Reactions were allowed to incubate for 1 h, washed to remove nonspecifically bound proteins, and eluted with SDS sample buffer. MW is molecular weight. The Western blot was probed with rat mAb 2B4 [23].

The ability of the mAbs to immunoprecipitate (IP) *E. coli*-expressed EBNA1 was tested. MAb resin was made by crosslinking the mAb to rProteinA-agarose beads. First, EBNA1 was overexpressed in *E. coli* and whole cell extract was prepared. The mAb resin was mixed with the soluble fraction of the whole cell extract, washed with lysis buffer followed by a more stringent wash with high salt buffer, and protein was eluted by SDS sample buffer and heating. Resin alone did not bind any EBNA1 protein, but all mAbs were able to IP EBNA1 (Fig. 3). MAb 3EB13 purification was not as strong when the salt in the second wash was increased to 500 mM NaCl, suggesting the interaction between this mAb and EBNA1 is weakened by higher salt.

### Polyol-responsiveness of mAbs

A unique property of a few mAbs, termed polyol-responsiveness, was first described in our lab [15], [16]. These mAbs maintain a high affinity and tight interaction with their antigen during loading and washing, but release under special gentle conditions.

To determine which mAbs have this characteristic, we performed a modified enzyme-linked immunosorbent assay (ELISA), termed ELISA-elution assay. This procedure follows a typical protocol for an ELISA assay, but adds a salt-polyol buffer incubation step. The primary mAb is released if the salt-polyol buffer reduces its affinity for antigen. Unbound primary mAb is washed away and detection of bound mAb by a secondary antibody is reduced.

**Figure 4:**
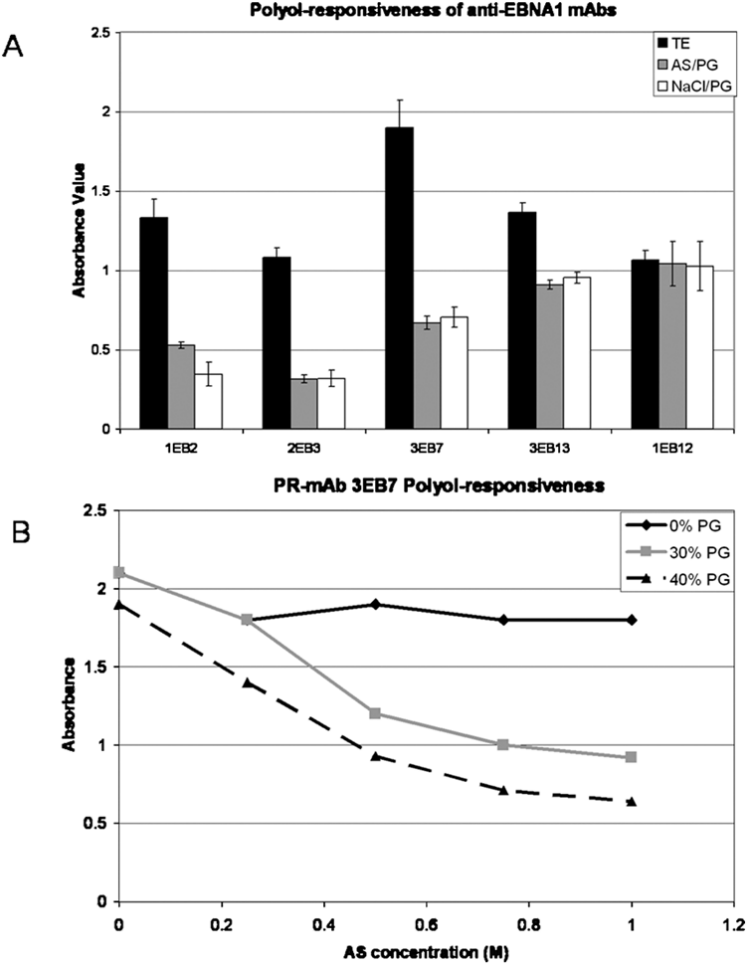
Polyol-responsiveness of anti-EBNA1 mAbs. A) ELISA-elution assay results to determine polyol-responsiveness. A standard ELISA protocol was performed with the addition of an intermediate step. After the anti-EBNA1 mAbs are bound to the EBNA1-coated well, either a control buffer (TE: 50 mM Tris-HCl, pH 7.9+0.1 mM EDTA) or a salt-polyol buffer (AS/PG: TE buffer+1 M ammonium sulfate+40% propylene glycol or NaCl/PG: TE buffer+1 M NaCl+40% propylene glycol) was added to the wells. MAbs that are polyol-responsive will have lower affinity for their antigen in the salt-polyol buffer which will result in a decrease in signal after the addition of a secondary antibody and the ELISA substrate. The experiment was done in triplicate. B) Detailed analysis of mAb 3EB7 response to various combinations of ammonium sulfate (AS) and propylene glycol.

The anti-EBNA1 mAbs were tested for polyol-responsiveness. MAbs 1EB2, 2EB3, 3EB7, and 3EB13 were found to have a reduced signal when incubated with a salt-polyol buffer containing either 1 M AS+40% PG or 1 M NaCl+40% PG (Fig 4A). MAb 1EB12 shows a typical ELISA-elution assay result of a mAb that is not polyol-responsive. In general, a mAb is considered polyol-responsive if the signal drops to 50% of the TE buffer signal when incubated with polyol buffer. The signal for PR-mAbs 1EB2, 2EB3, 3EB7, and 3EB13 dropped to 25%, 30%, 33% and 63% of the original signal, respectively. PR-mAb 3EB13 did not drop to the 50% threshold; however, our lab has shown that this decrease might still allow for efficient immunoaffinity purification (R. Burgess, personal communication). A detailed analysis of PR-mAb 3EB7 polyol-responsiveness is shown in Fig. 4B. An increase in salt alone did not decrease the signal significantly. PR-mAb 1EB2 is sensitive to salt alone, without any polyol.

As discussed previously [13], [19], we do not know the mechanism of the polyol/salt elution or the physicochemical properties that make certain mAbs polyol-responsive.

### EBNA1 immunoaffinity chromatography using PR-mAbs

**Figure 5:**
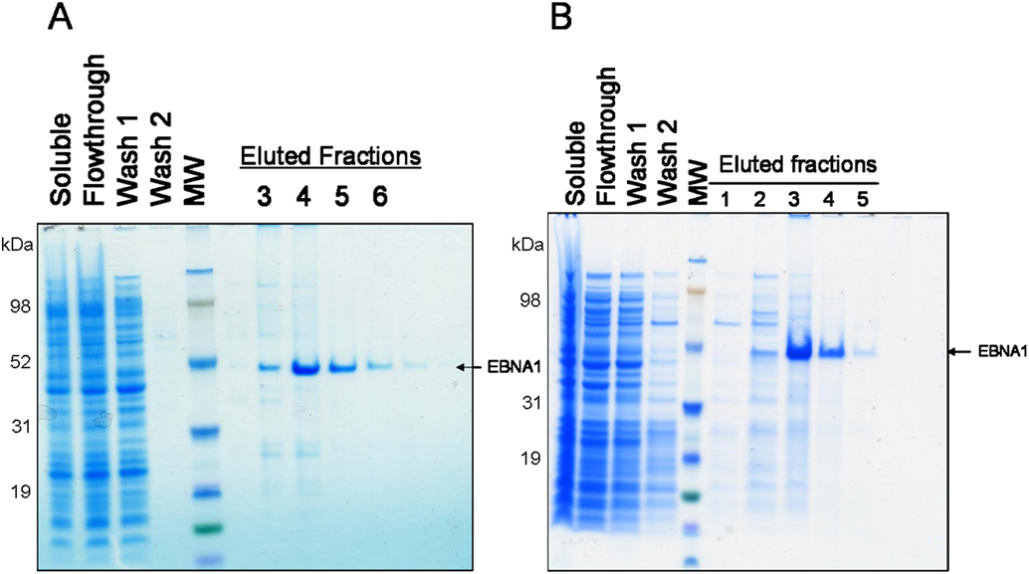
Immunoaffinity chromatography using PR-mAbs. The soluble fraction of whole cell extract from *E. coli* overexpressing EBNA1 was added to A) mAb 1EB2-Sepharose column and B) mAb 3EB7-Sepharose column, washed, and eluted with TE+0.75 M AS+40% PG. Coomassie blue stained gels are shown.

Utilizing PR-mAbs in immunoaffinity chromatography (IAC) allows for purification of active, nondenatured proteins and protein complexes. The utility of PR-mAbs 1EB2 and 3EB7 to purify EBNA1 from whole cell extract was tested. EBNA1 was overexpressed in *E. coli* and the whole cell extract was applied to a 4-ml column of the appropriate immunoaffinity resin. Both columns were washed with lysis buffer. 1EB2-Sepharose was then washed with 40% PG alone while 3EB7-Sepharose was washed with TE+0.5 M AS. 1EB2-Sepharose was not washed with the more stringent salt conditions due to its salt sensitivity. EBNA1 was eluted from both columns with TE+0.75 M AS+40% PG. Both purifications show an efficient single-step immunoaffinity chromatography procedure resulting in high yield and purity of EBNA1 (Fig. 5).

Since EBNA1 has no known independent enzymatic activity, it most likely carries out its numerous functions through interaction with other proteins. Using these PR-mAbs to gently elute EBNA1 complexes would provide a new, powerful method to elucidate EBNA1-associated proteins. This group of PR-mAbs covers three epitope groups. Use of a set of PR-mAbs against EBNA1 would allow better coverage of the protein and, presumably, recover a range of EBNA1 complexes that may have been missed when probing with only one mAb, due to possible competition between the mAb and certain interacting partners.
